# FRAX Calculated without BMD Resulting in a Higher Fracture Risk Than That Calculated with BMD in Women with Early Breast Cancer

**DOI:** 10.1155/2018/4636028

**Published:** 2018-10-04

**Authors:** Rizky Suganda Prawiradilaga, Victoria Gunmalm, Trine Lund-Jacobsen, Eva Wulff Helge, Charlotte Brøns, Michael Andersson, Peter Schwarz

**Affiliations:** ^1^Department of Nutrition, Exercise, and Sports, University of Copenhagen, Nørre Allé 51, 2200 Copenhagen N, Denmark; ^2^Faculty of Medicine, Bandung Islamic University, Tamansari No. 2, Bandung 40116, Jawa Barat, Indonesia; ^3^Department of Endocrinology, Rigshospitalet, Copenhagen, Blegdamsvej 9, 2100 Copenhagen Ø, Denmark; ^4^Department of Oncology, Rigshospitalet, Blegdamsvej 9, 2100 Copenhagen Ø, Denmark; ^5^Faculty of Health Sciences, University of Copenhagen, Blegdamsvej 3B, 2200 Copenhagen N, Denmark

## Abstract

**Background (and Purpose):**

The aim of this study was to investigate the importance of including the measurement of bone mineral density (BMD) in reliable fracture risk assessment for women diagnosed with early nonmetastatic breast cancer (EBC) before AI treatment if zoledronic acid is not an option.

**Material and Methods:**

One hundred and sixteen women with EBC were included in the study before initiating AI treatment. Most participants were osteopenic. The 10-year probability of hip fracture and major osteoporotic fracture was calculated with and without BMD based on clinical information collected at baseline using the fracture risk assessment (FRAX) tool. To compare data, the nonparametric tests were used.

**Results:**

There was a significant difference (p<0.001) in the number of high-risk and low-risk FRAX score of hip fracture between before and after including BMD values. The high-risk category decreased by 50.9%, while the low-risk category increased by 42.9%. In FRAX score of major osteoporotic the findings were similar (p<0.001): The high-risk and moderate-risk category decreased by 70.4% and 4.9%, respectively, while the low-risk category increased by 43.8% when including BMD value. When stratified by age, patients aged 65 years or older were at a significantly (p<0.001) higher risk of suffering a hip or major osteoporotic fracture, highlighting the importance of including BMD measurements in this age group.

**Conclusions:**

Our data support that DXA scanning of women with EBC should be performed to avoid overestimation of osteoporosis before AI treatment. It is particularly important in patients older than 65 years of age and when zoledronic acid is not an option.

## 1. Introduction

Breast cancer treatment has improved in Europe over the past decades with an 82% 5-year age-adjusted relative survival for women diagnosed between 2000 and 2007 [1]. However, breast cancer is still the number one cause of cancer-related death in Europe, although lung cancer as the number two cause of death in Europe is the most frequent cause of cancer death in Denmark. The currently expected levels of survival are improved in part due to breast cancer screening programs providing earlier detection and new improved adjuvant therapy options [2]. The decreased mortality rate is mainly seen among younger patients [2].

After initial treatment of breast cancer by surgery patients are often offered adjuvant treatments such as radiotherapy, chemotherapy, HER2-directed treatment, and antihormonal therapy for patients with estrogen receptor positive disease (approximately 75%). Adjuvant antihormonal treatment is for premenopausal patients usually 5-10 years on tamoxifen and for postmenopausal patients five years on the aromatase inhibitor (AI) [2]. The AIs improve both disease-free survival (DFS) and overall survival (OS)(OS compared with tamoxifen) [3].

Patients on therapy with tamoxifen do not increase their risk of bone loss whereas the AI-induced ovarian suppression of estrogen production increases the risk of bone loss and fracture [4]. It is widely known that women undergoing therapy with AI are recommended for supplementation with calcium and vitamin D. Thus far, a dual energy X-ray absorptiometry (DXA) scanning prior to AI therapy is recommended to avoid overestimation of osteoporosis.

Since 2015 it is recommended to administer zoledronic acid, a bisphosphonate, along with AI in early nonmetastatic breast cancer (EBC) [5]. Zoledronic acid is effective in fracture prevention in postmenopausal women with osteoporosis [6]. It is further known from the meta-analysis published by the Early Breast Cancer Trialists' Collaborative Group (EBCTCG) that some antineoplastic effect is gained from zoledronic acid [5]. Based on this double effect, it might be questioned if DXA-scanning is relevant at the beginning of treatment initiation with AI and zoledronic acid.

Since many breast cancer patients encounter treatment-related distress and findings indicate that women offered a DXA-scan refuse because of this stress, tools to support clinical decision-making regarding the treatment of patients with low bone mass are indeed needed [7, 8]. The risk assessment tool, fracture risk assessment (FRAX®), has been developed by the University of Sheffield and is widely used in the clinic [9]. FRAX integrates clinical risk factors: country, age, sex, weight, height, previous fracture, family fracture history, smoking, glucocorticoid treatment, rheumatoid arthritis, disease strongly associated with osteoporosis, and alcohol intake with or without assessment of bone mineral density (BMD) at the femoral neck, to evaluate the 10-year probability of hip fracture and major osteoporotic fractures (clinical spine, forearm, hip, or shoulder fracture).

In the present study, we evaluated the importance of age and BMD in reliable fracture risk assessment in a cohort of women diagnosed with EBC.

## 2. Methods

The target group for the present study was women diagnosed with EBC. In total, all 133 diagnosed women were recruited from May 2016 to May 2017 at the Department of Endocrinology, Rigshospitalet, Copenhagen, Denmark, for further evaluation. Twelve women were excluded due to previously diagnosed osteoporosis (T-score < -2.5) and/or ongoing treatment with antiresorptive medication (e.g., Alendronate), and five women were excluded due to missing data, resulting in a total of 116 women. All participants were subject to routine examination, anthropometric and BMD measurements.

### 2.1. Bone Mineral Density

BMDs were measured at the lumbar spine (LS) (the mean of L2-L4), femoral neck (FN), and total hip (TH) on both sides. DXA accurately determines two-dimensional BMD (g/cm^2^) and detects patients with fragile bones who are at an increased risk of incurring osteoporotic fractures [10].

The BMDs of LS (L2–L4) and both hips (TH and FN) were routinely measured using DXA (Hologic DiscoveryTM QDR Series scanner), and the trabecular bone scores were calculated afterward (TBSiNsightTM version 1.9.2.1, Med-Imaps). Skeletal sites with metal implants were excluded.

The same laboratory technician performed all analyses. The calibration was performed following the standard procedure. According to the manufacturer, the CV (coefficient of variation) of the total BMD is approximately 1% Europe H. Hologic Osteoporosis Assessment. Reference Manual. 2006; Document No. Man-00214.

### 2.2. Biochemistry

All blood samples were routinely obtained via venipuncture in the antecubital vein and processed at the central laboratory at Rigshospitalet, Denmark, for future analysis of the plasma concentrations of free calcium-ion, 25-OH vitamin D, parathyroid hormone, serum phosphate, alkaline phosphatase, bone-specific alkaline phosphatase, procollagen, and osteocalcin to ensure that no other bone disease or other disease was present.

### 2.3. FRAX

The 10-year probability (FRAX scores) of hip fracture or major osteoporotic fracture with or without BMD was calculated by fracture risk assessment tool country-specific for Denmark on the official website (https://www.sheffield.ac.uk/FRAX/) based on a participant's risk at the time of the DXA examination.

FRAX score of hip fracture ≥ 3% was defined as a high-risk category, and vice versa: if the score was below 3%, it was categorized as low-risk. Meanwhile, FRAX score of major osteoporotic fracture ≥ 20% was defined as a high-risk category, 10-19% as a moderate category, and <10% as a low-risk category [11].

### 2.4. Statistical Analysis

The statistical package for the social sciences (SPSS, version 24.0, IBM Corp., Armonk, NY, USA) was used for all statistical analyses. The nonparametric test was drawn to investigate differences between the FRAX score without BMD value and the FRAX score with BMD value. A p value ≤ 0.05 was considered statistically significant.

## 3. Results

The baseline clinical characteristics of the participants are presented in Table 1. The mean age was 64.1±11.2 years and the median was 64 years. The average LS, TH, and FN T-scores were in the osteopenic range (T-score between -1.0 and -2.5). The specific BMD measures are presented in Table 2. One participant was excluded due to incomplete data. As shown, only 2.6% of the participants were classified within the normal range when evaluating BMD at all sites. Osteopenia was seen in 64.4% (BMD T-score below -2.5) and 33% had osteoporosis (BMD T-score less than -2.5) in one or more sites.

Table 3 shows the 10-year probability of suffering a hip fracture. As shown, when adding BMD measurements to the FRAX score calculation, the number of patients in the high-risk category was reduced from 53 to 26 patients, a statistically significant decrease (relative risk reduction) of 50.9% (p<0.001). The low-risk category increased by 42.9%.

Table 4 presents the calculated 10-year probability of a major osteoporotic fracture with and without the BMD measurement included. When including BMD measurements in the FRAX major score calculation, the number of patients in the high-risk category significantly decreased by 70.4% from 27 to 8 patients (p<0.001), whereas the moderate risk category decreased by 4.9% from 41 to 39 patients. The low-risk category increased accordingly by 43.8% from 48 to 69 patients.

Tables 5 and 6 show the FRAX estimates with and without FN BMD included and stratified by age. As shown, when based on FRAX scores without the inclusion of FN BMD, patients aged 65 years or older were estimated to have a much higher risk of suffering a hip fracture and a major osteoporotic fracture than younger patients. Thus, 90.4% of the patients ≥65 years of age were in the high-risk category for hip fracture, compared to only six patients (9.4 %) in the lower age group, as shown in Table 5.

However, when including FN BMD measurements in the FRAX assessment, we observed a significant (p<0.001) decrease of 24 patients (51.1%) in the high-risk group among patients ≥65 years of age. For the patients <65 years of age (n=64) the trend was similar; there was a decrease in proportion by 50% from 6 to 3 patients, but the change was not statistically significant (p=0.453) since the low-risk group only changed 5.2% from 58 to 61 patients.

The same pattern was seen when calculating risk of major osteoporotic fracture, where all patients ≥65 years of age (n=52) qualified into the moderate- (n=25, 48.1%) or high-risk category (n=27, 51.9%), whereas none of the younger patients qualified into the high-risk category, 16 (25.0%) into the moderate, and 48 (75.0 %) into the low-risk category, as shown in Table 6.

Including FN BMD measurements in the risk assessment, a significant (p<0.001) decrease of 19 patients (70.4%) in the high-risk group among patients ≥65 years of age was seen. The moderate-risk category increased by eight patients (32%) and the low-risk category became 11 patients. When including FN BMD measurements for patients <65 years of age the moderate risk category decreased significantly (p=0.008) by ten patients (62.5%) and the low-risk group increased accordingly.

From the statistical analysis, there were highly and clinically relevant significant differences (p value <0.001) in the above FRAX scores between the two age groups.

## 4. Discussion

Our data demonstrate that the average estimates of fracture risk appear to be higher when BMD measurement was not included in the FRAX calculation in EBC women before starting AI treatment. The treatment with AI would even further increase the risk of misjudgment as AI treatment is not included in the FRAX algorithm. The results are in accordance with other studies showing that risk estimates without BMD are not as accurate in predicting fractures as FRAX calculated with BMD [12, 13]. In a newly published paper by Hamdy et al. they showed that FRAX calculated without BMD does not correctly identify Caucasian men with BMD evidence of osteoporosis [14]. The same observation might be expected if evaluated in postmenopausal women although not yet investigated.

Identification and treatment of EBC patients at high risk of sustained fractures based on the presence of clinical risk factors such as AI treatment are clinically relevant. However, assessment disregarding BMD may lead to a number of patients wrongly evaluated with high risk, who will be unnecessarily treated with medications that affect BMD but do not alter the underlying risk factors.

Our data shows that the number of EBC patients at fracture risk seems significantly lower than that calculated by FRAX without including BMD. In addition to this observation, the evidence of the use of FRAX in EBC is very sparse.

Leslie et al. [15] studied 36,730 women and 2,873 men, 50 years of age and older, from Manitoba, Canada, and showed that a FRAX designation of the high risk of fracture is commonly associated with a densitometric diagnosis of osteoporosis. In accordance with their conclusions, the results of our study demonstrate that the majority (65.4%) of patients with a FRAX designation of “high-risk” of any hip fracture over 10 years and all patients (100%) with a FRAX designation of “high-risk” of any major osteoporotic fracture over 10 years had at least one T-score measurement of -2.5 or lower if we include the BMD values in FRAX evaluation. Conversely, if we do not include the BMD values in FRAX evaluation, only 39.6% (n=21) of 53 patients with 10-year probability of hip fracture and 44.4% (n=12) of 27 patients with the 10-year probability of major osteoporotic fracture had at least one T-score measurement of -2.5 or lower. This means FRAX calculation without BMD values in postmenopausal women diagnosed with EBC was overestimated.

For the correct FRAX estimate, FN data are needed [https://www.sheffield.ac.uk/FRAX/]. However, it has previously been shown that even measurements of left hip vs. right hip affect the risk assessment, as up to a 9% difference in BMD has been reported between hips [16]. Taking this into account, it could be questioned whether the FRAX calculation should use the lowest femoral neck BMD measurement to increase the validity of FRAX.

Our data show a significant and clinically relevant difference in both the FRAX scores, i.e., an estimate of the risk of hip fracture and major osteoporotic fractures, between including and excluding BMD values. Due to this significant difference, the use of FRAX risk assessment without the inclusion of BMD data cannot be recommended in patients with EBC as FRAX overestimates the fracture risk, making the BMD inclusion highly clinically important in these patients. Our observations in postmenopausal EBC patients are in contrary to the study of Gadam et al. [17] who found identical fracture risk predictions with or without BMD included in the FRAX calculation in healthy postmenopausal women aged 50 years or older. It is possible that our EBC patients might differ from healthy postmenopausal women. However, EBC women evaluated before AI treatment might only differ due to surgery and chemotherapy treatment as no complications are found in our patient group. Therefore the observed difference between the present study and the study by Gadam et al. [17] is surprising.

Our results also highlight the importance of age, as we report a clinically relevant significant difference between age groups. The FRAX risk assessment in our study is highly influenced by age, which is in alignment with the data by the National Osteoporosis Group [18] and Gadam et al. [17]. However, Gadam et al. demonstrated that, in healthy postmenopausal women, age was the only significantly different risk factor between those with identical and different predictions [17].

Our findings support the study by Gourlay et al. [19] involving 4,957 postmenopausal women with normal BMD or mild osteopenia, which encouraged an increase in screening interval of BMD in postmenopausal women according to their BMD values. The FRAX score can be used as a screening instrument in postmenopausal women, and if the score reveals an increased risk for osteoporotic fracture, then a DXA scan can be acquired to get baseline BMD data preceding the treatment. The same should be recommended for EBC patients.

The standard care of EBC patients in Denmark now includes zoledronic acid treatment every six months for four years during AI treatment. The treatment is not based on bone loss protection but as adjuvant antineoplastic treatment of BC based on the study of the Early Breast Cancer Trialists' Collaborative group [5]. Zoledronic acid is very well known as the antiresorptive treatment of postmenopausal bone loss reducing the risk of bone fractures. However, due to side effects or to other unknown reasons, several EBC patients refuse zoledronic acid treatment. To avoid overestimation of the fracture risk the BMD evaluation and FRAX calculation may be highly recommended before other treatment options are considered in these cases. The algorithm of Hadji P et al. is therefore still recommended [4].

Without knowing the true or actual fracture in our small cohort of patients, we cannot determine which of the estimates of risk is more correct. This is the limitation of the present study.

## 5. Conclusion

Our data support that, before AI treatment, DXA screening of patients suffering from EBC should be performed for the correct estimate of fracture risk assessment. This is particularly important in patients older than 65 years of age and when zoledronic acid treatment is not an option.

## Figures and Tables

**Table 1:** Baseline characteristics of the participants.

Characteristics	(*n*=116)
Age (years)	64.1 ± 11.2
Height (cm)	164 ± 7
Weight (kg)	67.1 ± 13.2
BMI (kg/m^2^)	24.8 ± 4.4
BMD LS (g/cm^2^)	0.997 ± 0.167
T-Score LS	-1.6 ± 1.3
BMD left TH (g/cm^2^)	0.806 ± 0.108
T-Score left TH	-1.6 ± 0.8
BMD right TH (g/cm^2^)	0.817 ± 0.103
T-Score right TH	-1.5 ± 0.9
BMD left FN (g/cm^2^)	0.778 ± 0.111
T-Score left FN	-1.6 ± 0.8
BMD right FN (g/cm^2^)	0.773 ± 0.145
T-Score right FN	-1.6 ± 0.8

Data are presented as mean ± SD.

LS: lumbar spine; TH: total hip; FN: femoral neck.

**Table 2:** BMD measures at spine, total hip, and femoral neck.

BMD Risk Category	Spine (n=115)	Left Hip		Combined Spine & Left Hip^a,b,c^ (n=115)	p value between the groups^∗^
		Total Femoral (n=115)	Femoral Neck (n=115)		
Normal (WHO criterion [T-score ≥-1])	29 (25.2%)	31 (26.9%)	32 (27.8%)	3 (2.6%)	
Osteopenia (WHO criterion [T-score between -1 and -2.5])	58 (50.4%)	70 (60.9%)	67 (58.3%)	74 (64.4%)	<0.001
Osteoporosis (WHO criterion [T-score ≤-2.5])	28 (24.4%)	14 (12.2%)	16 (13.9%)	38 (33.0%)	

Data presented as *n (*%).

^*∗*^Statistical testing across group using Friedman test.

^a^Combined BMD LS and left TH vs BMD TS alone, p value <0.001 (post hoc Wilcoxon test).

^b^Combined BMD LS and left TH vs BMD left TH alone, p value <0.001 (post hoc Wilcoxon test).

^c^Combined BMD LS and left TH vs BMD Left FN alone, p value <0.001 (post hoc Wilcoxon test).

Other pairs which are not mentioned are not statistically significant.

**Table 3:** The 10-year probability of hip fracture. Without vs with using BMD on calculation.

**FRAX Score of hip fracture category **	Without BMD (n=116)	With BMD (n=116)	p value between FRAX Score without using BMD and with BMD^∗^
Low-risk (≤ 3%)	63 (54.3%)	90 (77.6%)	<0.001
High-risk (> 3%)	53 (45.7%)	26 (22.4%)	

*Data presented as n (*%*).*

^∗^ *McNemar test.*

**Table 4:** The 10-year probability of major osteoporotic fracture. Without vs with using BMD on calculation.

**FRAX score of major osteoporotic fracture category**	Without BMD (n=116)	With BMD (n=116)	p value between FRAX Score without using BMD and with BMD^∗^
Low-risk (<10%)	48 (41.4%)	69 (59.5%)	<0.001
Moderate-risk (10-20%)	41 (35.3%)	39 (33.6%)	
High-risk (>20%)	27 (23.3%)	8 (6.9%)	

*Data presented as n (*%*).*

^∗^ *Wilcoxon test.*

**Table 5:** The 10-year probability of hip fracture stratified by age.

		FRAX Scores of Hip Fracture w/o BMD (n=116)		FRAX Scores of Hip Fracture w/ BMD (n=116)	
		Low-risk	High-risk	Low-risk	High-risk
Age group^∗^	<65^a^ (n=64)	58(90.6)	6(9.4)	61(95.3)	3(4.7)
	≥65^b^ (n=52)	5(9.6)	47(90.4)	29(55.8)	23(44.2)

Data presented as n (%).

^*∗*^Statistical testing between the age groups using Chi Square test with Yates correction p <0.001.

^a^FRAX scores of hip fracture without BMD vs with BMD in below 65 years. p = 0.453 (McNemar test).

^b^FRAX scores of hip fracture without BMD vs with BMD in 65 years and above. p <0.001 (McNemar test).

**Table 6:** The 10-year probability of major osteoporotic fracture stratified by age.

		FRAX Scores of Major osteoporotic w/o BMD (n=116)			FRAX Scores of Major osteoporotic w/ BMD (n=116)		
		Low-risk	Moderate risk	High-risk	Low-risk	Moderate risk	High-risk
Age group^∗^	<65^a^ (n=64)	48(75.0)	16(25.0)	0(0.0)	58(90.6)	6(9.4)	0(0.0)
	≥65^b^ (n=52)	0(0.0)	25(48.1)	27(51.9)	11(21.2)	33(63.5)	8(15.4)

Data presented as n (%).

^*∗*^Statistical testing between the age groups using Chi Square for proportion (Pearson Chi Square) in group without BMD, Chi Square for trend test and Mann-Whitney test in group with BMD, p <0.001.

^a^FRAX scores of major osteoporotic fracture without BMD vs with BMD in below 65 years. p = 0.008 (Wilcoxon test).

^b^FRAX scores of major osteoporotic fracture without BMD vs with BMD in 65 years and above. p <0.001 (Wilcoxon test).

## Data Availability

The research article data used to support the findings of this study are available from the corresponding author upon request.
